# Evaluation of Organ Donation Attitudes in The Context of Health Literacy and Fatalism: A Cross‐Sectional Correlational Study

**DOI:** 10.1111/nhs.70145

**Published:** 2025-05-26

**Authors:** Duygu Öztaş, Sultan Türkmen Keskin, Yasemin Uğurlu, Şenay Akgün

**Affiliations:** ^1^ Department of Midwifery Ankara University Faculty of Nursing Ankara Turkey; ^2^ Department of Nursing, Faculty of Nursing Ankara University Ankara Turkey; ^3^ Zübeyde Hanım Faculty of Health Sciences, Department of Nursing Niğde Ömer Halisdemir University Niğde Turkey; ^4^ Faculty of Health Sciences, Department of Nursing Alanya Alaaddin Keykubat University Antalya Turkey

**Keywords:** fatalism, health belief model, health literacy, organ donation

## Abstract

The aim of the study is to investigate the relationships among health literacy (HL), fatalistic tendencies, and attitudes toward organ donation. A cross‐sectional correlational study design was employed. The sample consists of 1566 voluntary participants residing in four major cities in the Central Anatolian Region of Türkiye. Data were collected via an online form between August 1 and December 31, 2023, using the Organ Donation Attitude Scale, Health Literacy Scale‐14, and Fatalism Tendency Scale. Mediation analysis was performed utilizing Hayes' PROCESS Macro (Model 4). Of the participants, 72.8% demonstrated knowledge about organ transplantation, and 40.4% reported having considered organ donation after death. The analyses revealed that fatalistic tendencies explained 5.4% of the variance in humanity and moral conviction and 13.31% of the variance in negative attitudes. Additionally, HL partially mediated the relationship between fatalistic tendencies and attitudes toward organ donation. These findings suggest that addressing fatalism and improving HL could positively influence perceptions of organ donation.

**Summary**

- Organ shortage continues to be a significant global problem. Consequently, understanding the factors that shape public attitudes toward organ donation is essential for increasing donation intentions.
- This study revealed that high health literacy is associated with lower fatalistic tendencies and fewer negative attitudes toward organ donation.
- Furthermore, health literacy partially mediated the relationship between fatalistic tendencies and attitudes toward organ donation.
- Policymakers and health institutions could develop educational programs and campaigns that both address fatalistic beliefs and improve public health literacy, ultimately helping to increase organ donation rates.

## 1 Introduction

Organ donation and transplantation are essential medical interventions that save lives, enhance quality of life, and reduce both the burden and costs associated with long‐term patient care (Alshammari and Brown 2023; Alwahaibi et al. 2023). Despite these beneficial aspects of organ donation, global organ donation rates remain below the desired levels. According to 2022 data, the rate of organ donation from deceased donors is 6.87 per million worldwide, 16.85 per million in European countries, and only 3.38 per million in Türkiye (Global Observatory on Donation and Transplantation [GODT] 2022). Furthermore, the Turkish Tissue, Organ Transplantation and Dialysis Institute reported that in 2023, 1840 individuals lost their lives, and the families of only 305 deceased individuals (16.6%) consented to organ and tissue donation (Department of Tissue, Organ Transplantation and Dialysis Services 2023). These statistics highlight a significant gap in organ donation rates in Türkiye, underscoring the need for increased societal awareness and prioritization of organ donation.

## 2 Background

**FIGURE 1:**
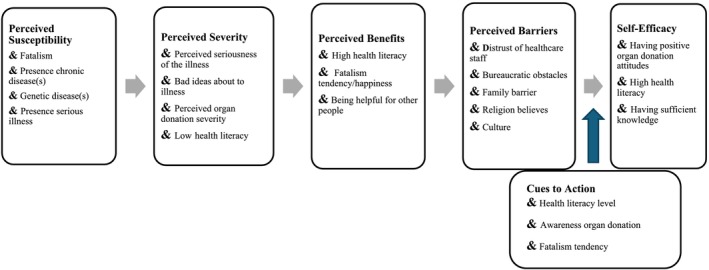
Factors affecting organ donation attitude according to Health Belief Model components (Adapted from Champion and Skinner 2008).

Organ donation is a multifaceted issue influenced by sociocultural dynamics, health literacy (HL), religious beliefs, and fatalism beliefs (Akbulut, Ozer, Gokce, et al. 2020; Alghamdi et al. 2023; Arıburnu et al. 2022; Belkebir et al. 2022; Can and Hovardaoglu 2017; Demirkiran et al. 2019; Fan et al. 2022; Taş et al. 2022; Zeng et al. 2023; Zhang et al. 2023). In this context, societal misperceptions regarding organ donation, religious and cultural barriers, and the lack of knowledge about the importance and process of donation affect individuals' decisions regarding organ donation (Soqia et al. 2023; Zhang et al. 2022). The Health Belief Model (HBM) provides a valuable theoretical framework for understanding and predicting attitudes toward organ donation by examining how individuals' health‐related beliefs and perceptions influence their decision‐making processes (Pradeep et al. 2023). According to the HBM, factors such as perceived susceptibility to needing an organ, perceived benefits of organ donation, and perceived barriers to becoming a donor collectively influence individuals' willingness to donate organs (Champion and Skinner 2008; Pradeep et al. 2023) (Figure 1). The model focuses on the subjective benefits and risks that individuals evaluate when considering organ donation behavior (Pradeep et al. 2023). In this context, attitudes toward organ donation can also be shaped positively and/or negatively according to different emotional and intellectual states of individuals. Positive attitudes are based on humanitarian and moral convictions that stem from viewing organ donation as a humanitarian act benefiting society, from values of self‐sacrifice, and from ethical considerations (Pradeep et al. 2023; Yazıcı Sayın 2016). These attitudes focus on the perceived benefits of organ donation for both the recipient and the donor's family, and they may feel a sense of satisfaction or fulfillment in contributing to the gift of life (Pradeep et al. 2023). However, individuals may also develop negative attitudes (NA) toward organ donation due to reasons such as medical negligence or fear of physical harm (Parisi and Katz 1986; Yazıcı Sayın 2016). These attitudes are consistent with the HBM, which helps explain health behaviors by considering factors such as perceived susceptibility, perceived benefits, and perceived barriers. In particular, assessments of individuals' beliefs, knowledge, attitudes, and behaviors regarding organ donation are important to increasing donation rates. Understanding these factors at the individual level can offer valuable insights into strategies for improving donation rates (Pradeep et al. 2023).

Fatalism, defined as the belief that all events are predetermined and inevitable, may significantly impact attitudes toward organ donation (Can and Hovardaoglu 2017). Individuals with a fatalistic perspective often perceive preventable diseases, injuries, and deaths as “normal” and attribute them to “fate.” Therefore, these individuals may believe that organ transplantation is unnecessary or even against divine will (Molzahn et al. 2004). In addition, individuals with a fatalistic perspective may be reluctant to obtain and use health‐related information (Turan and Çiftçi 2023). Research indicates that attitudes toward organ donation are shaped by individuals' fatalistic tendencies, spirituality, and perception of purpose in life (Demirkiran et al. 2019; Taş et al. 2022; Zhang et al. 2023; Soylu et al. 2022). Thus, examining the relationship between fatalism and attitudes toward organ donation can explain the extent to which societal beliefs about fatalism influence these attitudes.

HL is the ability of individuals to obtain, understand, evaluate, and use basic health information to make informed healthcare decisions (Liu et al. 2020). HL enhances an individual's ability to maintain optimal health outcomes by improving access to and the critical evaluation of health‐related information (Liu et al. 2020; Ward et al. 2019). Higher levels of HL are consistently associated with positive health behaviors and improved health outcomes, as demonstrated by numerous studies (Chisholm‐Burns et al. 2018; Palumbo et al. 2021). In addition, low HL is associated with delayed healthcare utilization, more severe disease progression, and poorer overall health outcomes (Aljassim and Ostini 2020). Furthermore, studies suggest that limited HL may hinder individuals' information‐seeking behaviors, potentially leading them to develop more fatalistic beliefs regarding health and illness (Kobayashi and Smith 2016; Yildirim et al. 2025). These findings indicate the importance of HL as a critical tool for acquiring knowledge and fostering informed attitudes. In the context of organ donation, HL significantly influences societal beliefs and attitudes (Zeng et al. 2023; Zhang et al. 2022). Studies have shown that general knowledge about organ donation is limited, and public attitudes are often inconsistent (Alshammari and Brown 2023; Soylu et al. 2022; Şenyuva 2022; Tontus 2020). Additionally, low HL contributes to a lack of understanding about organ donation, perpetuating misconceptions and potentially fostering NA. Therefore, improving HL is important to increase public awareness of organ donation and encourage positive attitudes toward it.

Organ donation is a voluntary decision, and increasing awareness and participation in organ donation requires effective interventions. These interventions should focus on modifiable factors that influence individuals' perceptions, and they should be tailored to the specific characteristics of the target population (Alshammari and Brown 2023; Salim 2014). Evaluating the impact of fatalism and HL on organ donation is crucial for developing targeted public intervention programs. However, despite their significance, there is a notable gap in the literature regarding the interaction between fatalism, HL, and attitudes toward organ donation. While previous studies have explored various individual factors that affect organ donation, there remains a lack of research examining the specific roles of fatalism and HL, especially in the context of Turkish society. The aim of this study is to investigate the relationships between attitudes toward organ donation, HL, and fatalistic tendencies. The hypotheses (H) of the study are as follows:

Hypothesis 1 *Fatalistic tendencies are negatively associated with health literacy*.

Hypothesis 2 *Fatalistic tendencies are negatively associated with humanitarian and moral belief attitudes toward organ donation*.

Hypothesis 3 *Fatalistic tendencies are positively associated with negative attitudes toward organ donation*.

Hypothesis 4 *Health literacy is positively associated with humanitarian and moral belief attitudes toward organ donation*.

Hypothesis 5 *Health literacy is negatively associated with negative attitudes toward organ donation*.

Hypothesis 6 *Health literacy mediates the relationship between fatalistic tendencies and attitudes toward organ donation*.

## 3 Methods

### 3.1 Study Design and Participants

The research was conducted using a cross‐sectional correlational design. The Strengthening the Reporting of Observational Studies in Epidemiology (STROBE) checklist was used in the study. The sample comprised 1566 voluntary participants residing in Türkiye. The study population consisted of 12,896,255 individuals living in the Central Anatolian Region of Türkiye (T.C. Ministry of Internal Affairs 2021).

The required sample size (*n*) was calculated as 385 participants using the formula for a known population size (*N*), with a 95% confidence interval and a 5% margin of error (Dean et al. 2013). The procedure for determining the sample size is described below:

$$\begin{array}{c} n=\left[N\times t^{2}\times p\times q\right]/\left[d^{2}\times \left(N-1\right)+t^{2}\times p\times q\right]\\ \;=\left[12,896,255\times {\left(1.96\right)}^{2}\times 0.5\times 0.5\right]\\ \;/\left[{\left(0.05\right)}^{2}\times \left(12,896,255-1\right)+{\left(1.96\right)}^{2}\times 0.5\times 0.5\right]=385 \end{array}$$

Convenience sampling method was used to select participants. Invitations were distributed through online platforms including social media channels (Facebook, Instagram, X) and WhatsApp groups to residents of four major cities (Ankara, Konya, Kayseri, and Eskişehir) in the Central Anatolian Region. A total of 7810 individuals were invited to participate, and 1705 responded (response rate to invitation: 21.8%). Of these, 139 participants were excluded due to incomplete responses and duplicate submissions resulting in a final sample size of 1566 voluntary participants. The research sample consisted of Turkish citizens aged 18 and above who willingly consented to take part in the investigation.

### 3.2 Instruments

#### 3.2.1 General Information Questionnaire

A sociodemographic form consisting of 11 questions was utilized to collect data on participants' age, education, gender, employment status, number of children, and other relevant variables.

#### 3.2.2 Organ Donation Attitudes Scale

The measurement instrument, first developed by Parisi and Katz (1986), was subsequently validated for Turkish culture by Yazıcı Sayın (2016). Demonstrating good reliability (Cronbach's *α* = 0.88), this assessment tool incorporates 40 items structured in two distinct dimensions using a six‐point Likert format. The first dimension, consisting of 20 items, evaluates positive attitudes, emphasizing humanity and moral conviction related to organ donation. The second dimension, consisting of 20 items, assesses NA, particularly concerns about medical negligence and physical harm. Scores for positive and negative dimensions are calculated separately, offering a comprehensive evaluation of participants' attitudes toward organ donation. In this study, the Cronbach's alpha coefficients were found to be 0.921 for the humanity and moral conviction dimension of the scale and 0.932 for the NA dimension.

#### 3.2.3 Health Literacy Scale (HLS‐14)

HLS‐14 was originally developed by Suka et al. (2013) and later adapted to Turkish culture by Türkoğlu and Kılıç (2021). The scale comprises 14 items distributed across three dimensions, designed to assess HL. A five‐point Likert type was used. Assessment scores span from a minimum of 14 to a maximum of 70, where elevated scores reflect enhanced HL competency. The Cronbach's alpha value of scale is 0.85 (Türkoğlu and Kılıç 2021). In this study, the Cronbach's alpha coefficient for the scale was found to be 0.890.

#### 3.2.4 Fatalism Tendency Scale (FTS)

The FTS, developed by Kaya and Bozkur (2015), comprises 24 items divided into four sub‐dimensions. A five‐point Likert type was used. The total fatalism tendency (FT) score is calculated by summing the scores of all sub‐dimensions, where higher scores indicate a stronger inclination toward fatalism. The Cronbach's alpha value of scale is 0.86 (Kaya and Bozkur 2015). In this study, the Cronbach's alpha coefficient for the scale was found to be 0.835.

### 3.3 Data Collection

Data were collected online between August 1 and December 31, 2023. The invitation process was carried out through social media platforms. Local ethnic and regional platforms related to Central Anatolia were selected in particular. With the permission of group administrators, participation invitation links were sent to potential participants three times intermittently via the private messaging systems of these platforms. In order to ensure that the messages were not perceived as spam, the purpose of the study, participation conditions, and explanations were clearly stated in the invitations. Participants were informed about the study through a consent statement displayed on the first page of the online survey. A process was implemented where participants could only participate voluntarily and with informed consent. Consent was obtained for participation, and the confidentiality of personal data was fully protected. The average completion time for this survey is estimated to be approximately 20–25 min. Only those who gave informed consent were able to proceed to complete the survey.

Access to the online survey form was closed after December 31, 2023. All data were stored in a password‐protected Google Drive account accessible only to the researchers, and no personally identifiable information was collected during this process.

### 3.4 Statistical Analysis

The data analysis was conducted utilizing IBM SPSS 22.0 software package. Demographic data were analyzed using frequencies and percentages, while scale scores were evaluated through means and standard deviations. Pearson correlation analysis and linear regression were used to examine the relationships between continuous variables. Mediation effects were tested through hierarchical regression analyses using the PROCESS Macro Model 4 (Hayes 2017) in SPSS. To investigate the mediating mechanisms, the analysis employed bootstrap resampling with 5000 iterations. Bootstrapping was selected because it does not impose normality assumptions on the sampling distribution of indirect effects, provides more accurate confidence intervals (Preacher and Hayes 2008), and offers higher statistical power while maintaining appropriate Type I error control compared to traditional methods (Mackınnon et al. 2004). The statistical procedure quantified the total, direct, and indirect effects within the proposed model, with significance determined at the 95% confidence level.

## 4 Results

The mean age of the participants was 34.47 ± 17.90 years. Among the participants, 66.5% were women, 63.0% were single, 76.3% held a bachelor's degree, and 63.2% were without children. Additionally, 83.3% reported having no chronic diseases, 72.8% possessed knowledge of organ transplantation, and 41.1% received information about organ transplantation from television. Regarding organ donation, 40.4% of the participants stated that they had considered organ donation after death. However, 44.7% reported that they had not considered organ donation after death, with 48.1% indicating that it had not been a prior consideration for them (Table 1).

**TABLE 1:** Demographic characteristics of participants (*N* = 1556).

Groups	Frequency (*n*)	Percentage (%)
Age Ort = 34.470 ± 17.90 (Min = 18; Maks = 85)		
Gender		
Female	1034	66.5
Male	522	33.5
Marital status		
Married	576	37.0
Single	980	63.0
Education background		
Literate	29	1.9
Primary school	132	8.5
High school	207	13.3
Bachelor's degree and above	1188	76.3
Having children		
No	984	63.2
Yes	572	36.8
Chronic disease		
Yes	260	16.7
No	1296	83.3
Information on organ transplantation		
Yes	1132	72.8
No	424	27.2
* Where did he/she get information about organ transplantation?		
Healthcare worker	301	26.6
Family	96	8.5
Internet	146	12.9
Television	465	41.1
Newspaper	29	2.6
Other	246	21.7
Presence of organ donation card		
Yes	56	3.6
No	1500	96.4
Considering donating organs after death		
Yes	628	40.4
No	696	44.7
Undecided	231	14.9
* Reasons for not donating organs after death		
I have never thought about this	749	48.1
There is no specific reason	239	15.4
I don't feel ready	236	15.2
My health conditions are not suitable	115	7.4
I don't want my bodily integrity to be damaged	108	6.9
I don't find it religiously appropriate	69	4.4
I don't have enough information about organ donation	51	3.3
My family is against	25	1.6
I think my organs will be used for commercial purposes	15	1.0
I don't trust healthcare workers	9	0.6
I don't know where to apply	6	0.4

* Multiple selected items.

Table 2 presents the descriptive statistics for the scale scores. The mean HL score was 52.84 ± 9.68, the mean FT score was 62.46 ± 12.60, the mean humanity and moral conviction score was 99.09 ± 15.74, and the mean NA score was 56.61 ± 18.45. The results of the correlation analysis revealed that FT and NA were negatively correlated with HL (*r* = −0.227; r = −0.314, *p* < 0.001, respectively). In contrast, humanity and moral conviction were positively correlated with HL (*r* = 0.170, *p* < 0.001).

**TABLE 2:** Descriptive statistics and correlation for the study variables' scores (*N* = 1556).

	1	2	3	4
1. Fatalism tendency (FT) total	1.000			
2. Health literacy (HL) total	−0.227**	1.000		
3. Humanity and moral conviction (organ donation attitudes) (HMC)	−0.231**	0.170**	1.000	
4. Negative attitude (Organ donation attitudes) (NA)	0.365**	−0.314**	−0.448**	1.000
Mean score **±** SD	62.46 ± 12.60	52.84 ± 9.68	99.09 ± 15.74	56.61 ± 18.45
Min‐max score	24–97	14–70	30–120	20–120
Cronbach's alpha	0.835	0.890	0.921	0.932
Skewness	−0.041	−0.477	−1.084	0.430
Kurtosis	0.121	0.481	1.277	−0.408

Abbreviation: SD, standard deviation.

** *p* < 0.001.

**FIGURE 2:**
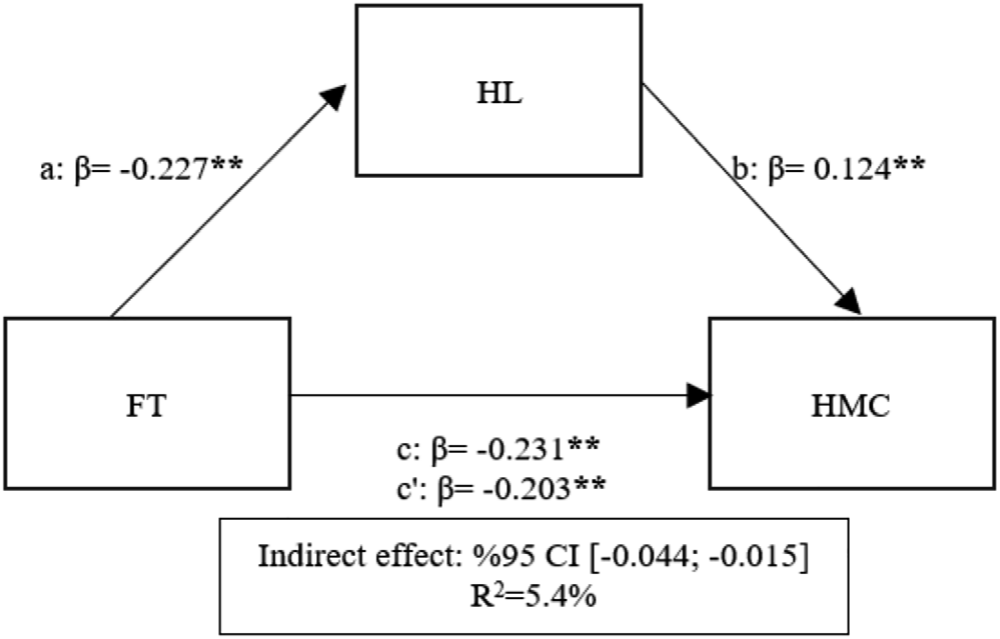
Mediation of HL in the relationship between FT and HMC.

The mediation analysis revealed that FT accounted for 5.4% of the total variance in Humanity and Moral Conviction (HMC) (*F* = 87.903; *p* < 0.05; see Table 3). A significant negative relationship was observed between FT and HL (path a) (*β* = −0.227; *p* < 0.05), providing support for Hypothesis 1 (H1). Similarly, the relationship between FT and HMC (path c) was also negative and significant (*β* = −0.231; *p* < 0.05), lending support to Hypothesis 2 (H2). The link between HL and HMC (path b) was positive and significant (*β* = 0.124; *p* < 0.05), supporting Hypothesis 4 (H4). Furthermore, the confidence interval for the indirect effect (95% CI [−0.044; −0.015]) did not include zero, indicating a statistically significant mediation. However, the indirect effect was notably smaller than the direct effect. Specifically, the relationship between FT and HMC decreased slightly from *β* = −0.231 (path c) to *β* = −0.203 (path c′) when HL was introduced, suggesting that HL acts as a partial mediator, with a relatively small effect size compared to the direct effect (Figure 2).

**TABLE 3:** Mediating effects of health literacy between fatalism tendency and humanity and moral conviction (*N* = 1556).

Dependent variable	Independent variable	*β*	Standart error	*t*	*p*	Bootstrap 95% CI	
						LLCI	ULCI
HL	FT (a)	−0.227	0.019	−9.174	0.000**	−0.211	−0.137
*R* = 0.227; *R* ^2^ = 0.051; *F* = 84.163; *p* = 0.000**							
HMC	FT (c)	−0.231	0.031	−9.376	0.000**	−0.350	−0.229
*R* = 0.231; *R* ^2^ = 0.054; *F* = 87.903; *p* = 0.000**							
HMC	FT (c’)	−0.203	0.031	−8.079	0.000**	−0.315	−0.192
	HL (b)	0.124	0.041	4.948	0.000**	0.122	0.283
*R* = 0.261; *R* ^2^ = 0.068; *F* = 56.859; *p* = 0.000**							
Total impact		−0.289	0.031	−9.376	0.000**	−0.350	−0.229
Direct impact		−0.254	0.031	−8.079	0.000**	−0.315	−0.192
Indirect impact		−0.028	0.007	—	—	−0.044	−0.015

Abbreviations: FT, fatalism tendency; HL, health litaracy; HMC, humanity and moral conviction; LLCI, lower limit confident interval; ULCI, upper limit confident interval; St. *β*, standardized regression coefficient; *β*, regression coefficient.

** *p* < 0.001.

**FIGURE 3:**
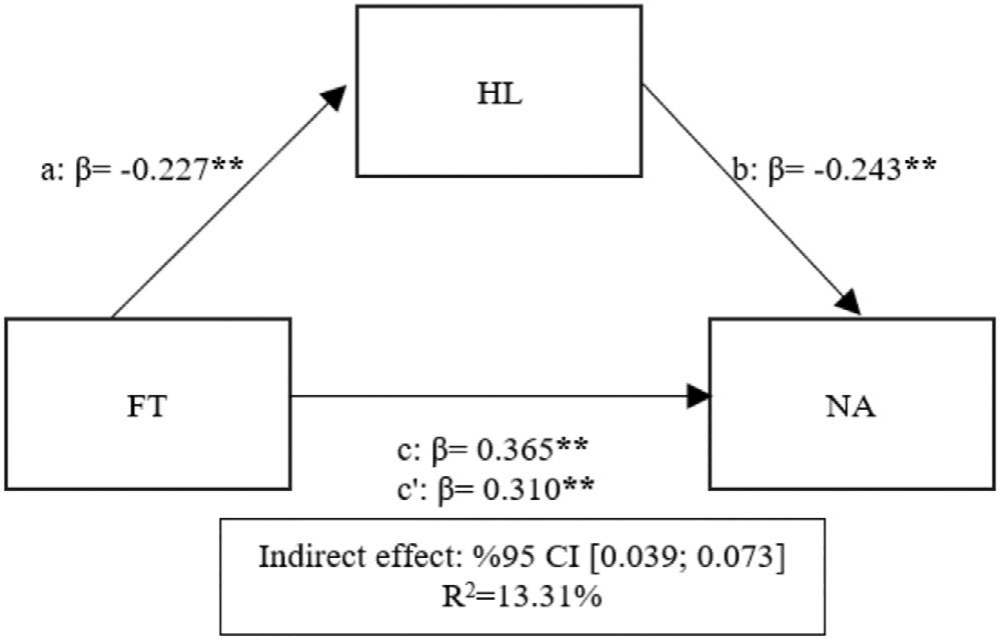
Mediation of HL in the relationship between FT and NA.

The analysis indicated that FT explained 13.31% of the total variance in NA (*F* = 238.557; *p* < 0.05; Table 4). A positive and significant relationship was found between FT and NA (path c; *β* = 0.365; *p* < 0.05), providing support for Hypothesis 3 (H3). Additionally, HL exhibited a negative and significant relationship with NA (path b; *β* = −0.243; *p* < 0.05), lending support to Hypothesis 5 (H5). The indirect effect was statistically significant (95% CI [0.039, 0.073]), although it was substantially smaller than the direct effect. Specifically, the relationship between FT and NA decreased from *β* = 0.365 (path c) to *β* = 0.310 (path c′) when HL was introduced, indicating that HL functions as a partial mediator with a modest effect size relative to the direct effect. These findings support Hypothesis 6, suggesting HL's mediating role in the relationship between FT and attitudes toward organ donation, although the mediation effects are relatively weak compared to the direct effects of fatalistic tendencies (Figure 3).

**TABLE 4:** Mediating effects of health literacy between fatalism tendency and negative attitudes (*N* = 1556).

Dependent variable	Independent variable	*β*	Standart error	*t*	*p*	Bootstrap 95% CI	
						LLCI	ULCI
HL	FT (a)	−0.227	0.019	−9.174	0.000**	−0.211	−0.137
*R* = 0.227; *R* ^2^ = 0.051; *F* = 84.163; *p* = 0.000**							
NA	FT (c)	0.365	0.035	15.445	0.000**	0.466	0.602
*R* = 0.365; *R* ^2^ = 0.133; *F* = 238.557; *p* = 0.000**							
NA	FT (c’)	0.310	0.034	13.199	0.000**	0.386	0.521
	HL (b)	−0.243	0.045	−10.376	0.000**	−0.551	−0.376
*R* = 0.435; *R* ^2^ = 0.189; *F* = 181.292; *p* = 0.000**							
Total impact		0.534	0.035	15.445	0.000**	0.466	0.602
Direct impact		0.453	0.034	13.199	0.000**	0.386	0.521
Indirect impact		0.055	0.009	—	—	0.039	0.073

Abbreviations: FT, fatalism tendency; HL, health litaracy; LLCI, lower limit confident interval; NA, negative attitudes; St. *β*, standardized regression coefficient; ULCI, upper limit confident interval; *β*, regression coefficient.

** *p* < 0.001.

## 5 Discussion

This study examined the relationship between individuals' attitudes toward organ donation and their fatalistic tendencies, as well as the mediating role of HL in this relationship. Results revealed that fatalistic tendencies were significantly associated with attitudes toward organ donation, with HL playing a partial mediating role in this relationship. All hypotheses of the research were statistically supported.

The current study revealed that the participants demonstrated a high level of HL. This finding is notable in light of previous research, as studies conducted in Türkiye have generally indicated low HL levels among the Turkish population (Aygun and Cerim 2021; Yiğitalp et al. 2021). This discrepancy may be explained by the fact that the majority of participants were women and held bachelor's degrees. Numerous studies have highlighted the significant roles of education level and gender in determining HL (Aljassim and Ostini 2020; Clouston et al. 2017; Lee et al. 2015; Sapbamrer et al. 2024; Sun et al. 2022). Specifically, being female and having a higher level of education are associated with better HL (Aljassim and Ostini 2020; Clouston et al. 2017; Lee et al. 2015; Svendsen et al. 2020). The results of the present study align with these findings.

Beyond the main hypotheses of the research, it is a noteworthy additional finding that approximately half of the participants obtained their knowledge about organ donation from television. This finding suggests that the media may play an important role in shaping individuals' health awareness. Parandeh Afshar et al. (2022) noted that higher media literacy may lead to increased HL, a result that aligns with the findings of the present study. Specifically, in our research, health information was primarily obtained through television. This finding is consistent with Belkebir et al. (2022), who found that mass media (television, internet, newspapers, etc.) is a key source of information about organ donation. In contrast, Santos et al. (2018) reported that younger individuals tend to prefer internet sources over traditional media, while Parandeh Afshar et al. (2022) found that most people first receive health information from the internet, followed by health providers. These differences may be attributed to the nature of the topic. Since organ donation is not a widely accepted or frequently discussed topic in Turkish society, information about organ donation obtained through television may be incidental.

In the present study, the level of FT was not high. Fatalism is a significant factor influencing health behaviors. Several studies have shown that a stronger tendency toward fatalism is associated with negative health behaviors, such as avoiding screening tests and maintaining an irregular diet. These studies also suggest that education level is a key determinant of fatalism (Kim and Lwin 2021; Öncü et al. 2021; Özer et al. 2021). Despite the generally high levels of fatalism in Türkiye, with most individuals exhibiting a strong fatalistic tendency (Çarkoğlu and Kalaycıoğlu 2009), the current study found that the FT was not particularly high. Moreover, these findings are inconsistent with previous research (Orhan 2017; Öncü et al. 2021; Özer et al. 2021). This discrepancy may be explained by the education level of the participants, as Orhan (2017) suggested that a higher level of education is associated with a lower tendency toward fatalism.

Individuals' humanitarian and moral attitudes toward organ donation encompass positive feelings such as appreciating the humanitarian benefits that organ donation provides to society and recognizing the moral value of this act. Conversely, negative emotions such as concerns about medical risks associated with the organ donation process, anxiety about bodily integrity being compromised, or fears of receiving inadequate healthcare reflect the NA that individuals develop toward organ donation (Parisi and Katz 1986; Yazıcı Sayın 2016). In this study, HMC attitudes were found to be high, whereas NA were found to be moderate. Regarding demographic characteristics, the majority of participants were female, single, and had a bachelor's degree, along with knowledge about organ transplantation. These results are consistent with findings from other studies (Alghamdi et al. 2023; Taş et al. 2022; Zhang et al. 2023). Alwahaibi et al. (2023) reported that positive attitudes toward organ donation are associated with a high level of organ donation knowledge. Fan et al. (2022) found a positive correlation between organ donation knowledge and attitudes toward donation. Similarly, Lim et al. (2020) indicated that a higher education level is a significant factor for positive organ donation attitudes. The study's findings may be related to explain by the high education level, high HL, and lower FT observed in the current study.

In the study, a positive relationship was identified between the HMC dimensions of organ donation and HL, and a negative relationship with FT. Similarly, a negative relationship was found between NA toward organ donation and HL, while a positive relationship was observed with FT. These findings suggest that FT is negatively related to attitudes toward organ donation, whereas HL is positively related to them.

HL may be conducive to participation in donation processes by aiding individuals in understanding the procedures, benefits, and critical needs associated with organ donation. In this regard, HL may play a crucial role in raising awareness, dispelling myths, and improving understanding of the donation process (Chakrabarty and Mishra 2024; Zeng et al. 2023; Zhang et al. 2023). However, our findings suggest that this role may be more modest than previously thought when considering the stronger direct influence of fatalistic beliefs.

The literature highlights that cultural and religious barriers to organ donation often include beliefs that illness is a manifestation of divine will and the influence of fatalism (Wray et al. 2023). If an individual holds fatalistic views stemming from their religious beliefs, they may perceive organ donation as interfering with or altering the natural order established by God or fate. According to Islamic beliefs, organ donation is not forbidden; in fact, it is considered beneficial for saving lives (Demirkiran et al. 2019). However, despite the absence of religious objections to organ donation, many individuals remain hesitant, particularly when it comes to cadaveric organ donation in Türkiye. A study conducted in Türkiye also found that the need for organ donation by family members or close relatives plays a critical role in an individual's willingness to become a donor (Akbulut, Ozer, Gokce, et al. 2020). This may be explained by the fact that when a person chooses to donate their organs while living, they maintain control over their body. Akbulut, Ozer, Firinci, et al. (2020) discovered that most Islamic religious officials do not endorse organ donation in the future (Akbulut, Ozer, Firinci, et al. 2020). In this context, HL can influence individuals' ability to make autonomous health‐related decisions and mitigate the impact of traditional beliefs (Smith et al. 2019; Wray et al. 2023). The study further revealed that HL partially mediates the relationship between fatalistic tendencies and attitudes toward organ donation, although the magnitude of this effect was relatively small. Mediation analyses showed that fatalistic tendencies primarily influence attitudes directly, with HL playing a statistically significant but limited role. While improving HL can enhance attitudes toward organ donation (Zeng et al. 2023), the findings suggest that fatalistic beliefs play a stronger role through direct pathways, indicating that addressing fatalism directly may be more effective. Therefore, comprehensive approaches targeting both HL and fatalistic beliefs could be effective in promoting organ donation.

### 5.1 Strengths and Limitations

The strength of this study lies in its examination of organ donation attitudes within the framework of fatalism and HL. Additionally, the study addresses a timely and socially relevant issue, with a large sample size that enhances the generalizability of the findings. However, there are several limitations to this study. First, the study's sample was geographically limited to four major cities within the Central Anatolia region, restricting the generalizability of results to the broader Turkish population. Moreover, the sample distribution was disproportionately represented by female participants and individuals with higher education levels, which may limit the generalizability of our findings and make it difficult to draw definitive conclusions about individuals with different sociodemographic characteristics. Furthermore, the recruitment of participants on a voluntary basis may have resulted in findings being limited to individuals predisposed to volunteer responses, excluding those who did not participate in the study.

Second, methodological limitations should be considered. The data relied on self‐reports, which may lead to response biases. The length of the survey may have made it difficult for participants to fully understand and respond accurately. Additionally, participants' anonymity was preserved, and no personal information (e.g., email addresses or IP addresses) was collected. Therefore, it was not possible to verify whether the questions were completed within the specified average time or to completely prevent duplicate responses. These limitations should be considered when interpreting the findings of this study.

Finally, a significant limitation of this study is that mediation analyses do not establish causality. While findings provide evidence supporting the specified mediation relationships among variables, alternative models remain possible. Future research could use more comprehensive analytical methods and qualitative approaches to further examine the complex relationships among variables and provide deeper insights that address these limitations.

## 6 Conclusion

This study found that both HL and fatalism are associated with attitudes toward organ donation. Moreover, statistical analysis suggests that HL partially mediates the statistical relationship between fatalism and attitudes toward organ donation. In light of these findings, healthcare institutions, transplant coordinators, and public health nurses might consider collaborating with religious leaders to design educational programs that address fatalistic beliefs and HL, which are related to organ donation attitudes. Additionally, future research should explore other factors that may be associated with attitudes toward organ donation and fatalism.

## 7 Relevance of Clinical Practice

Religious beliefs are quite important and effective in sustaining life. One of these beliefs, the fatalistic approach, can be a strong determinant in individuals making decisions about their own bodies and health. However, fatalistic attitudes can be changed with accurate and effective education programs aimed at increasing HL. As a result, NA in individuals toward issues such as organ donation can be transformed. In this context, it is of great importance for healthcare professionals and civil society organizations operating in this field to develop intervention programs aimed at eliminating information deficiencies and raising awareness regarding organ donation. Collaborating with religious leaders in the development and implementation of such intervention programs can increase the acceptability and impact of the programs. Thus, HL can be improved, fatalistic attitudes reduced, and social support for organ donation strengthened.

## Author Contributions

**Duygu Öztaş:** writing – review and editing, writing – original draft, validation, supervision, methodology, formal analysis, data curation, conceptualization, visualization, investigation. **Sultan Türkmen Keskin:** writing – review and editing, writing – original draft, validation, supervision, methodology, formal analysis, data curation, conceptualization, investigation. **Yasemin Uğurlu:** writing – review and editing, writing – original draft, methodology, formal analysis, data curation, investigation, conceptualization. **Şenay Akgün:** writing – review and editing, writing – original draft, methodology, formal analysis, data curation, conceptualization.

## Ethics Statement

Approval for the research was obtained from the Niğde Ömer Halisdemir University Non‐Interventional Clinical Research Ethics Committee (approval date: 05.07.2023, reference number: E‐86837521‐050.99‐381 213).

## Consent

Written permission to use the scales was acquired via email from the respective authors. The first page of the online survey included an informed consent document outlining the study details. Only those who provided consent were permitted to proceed to the subsequent pages of the survey.

## Conflicts of Interest

The authors declare no conflicts of interest.

## Supporting information


**Data S1.** Supporting Information.

## Data Availability

The data that support the findings of this study are available from the corresponding author upon reasonable request.
