# Supplementary material for: Evaluation of Organ Donation Attitudes in The Context of Health Literacy and Fatalism: A Cross‐Sectional Correlational Study
Source: Nurs Health Sci. 2025 May 26;27(2):e70145. doi: 10.1111/nhs.70145 (PMC12105880; doi:10.1111/nhs.70145)
Supplement: Supplementary file 1 — Data S1. Supporting Information. [file NHS-27-e70145-s001.pdf]

DATA COLLECTION FORM

Dear Participant,

We invite you to the research titled "Organ Donation: Evaluation in the Context of Health Literacy and Fatalism". The purpose of this research is to determine the relationship between the health literacy levels and fatalism views of individuals in society in their attitudes towards organ donation.

This study is a scientific research and the answers you provide will only be used as research data and will definitely not be shared with third parties. No fee will be requested from you during the research process and you will not be paid any fee. The research does not pose any risk or harm to you.Participation in the study is completely voluntary. You can withdraw from the study at any time without any negative consequences. This survey consists of 4 sections: Demographic information (12 questions), Organ Donation Attitude Scale (40 questions), Health Literacy Scale (14 questions), Fatalism Tendency Scale (24 questions). The survey will take approximately 20-25 minutes to complete.

This research has been approved by the Ethics Committee of XXXX (approval date: 05.07.2023, reference number: E-86837521-050.99-381213). In order to participate in the research, you must indicate your answers by marking the appropriate options below the questions.

If you have any questions about the research, you can contact:
Dr. Duygu ÖZTAŞ
Email: duygu.oztasQankara.edu.tr

□ I have read and understood the information provided above, and I voluntarily agree to participate in this research.

Part 1: DESCRIPTIVE CHARACTERISTICS

1. City you live in (……………)

2. Your age:

3.Gender	A) Famale	B) Male			
4. Marital status	A) Married	B) Single			
5. Educational status	A) Illiterate	B) Literate	C) Primary education	D) High school	E) University and above
6. Number of children, if any	………………… (please specify)				
7. Do you have a chronic disease? If yes, please specify	A) Yes (…………..)	B) No			
8. Do you have knowledge about organ transplantation?	A) Yes	B) No			
9. If your answer to the previous question is yes, where did you get this information from?	A) Healthcare worker	B) Television	C) Internet D) Family environment	D) Newspaper	E) Other
10. Do you have an organ donation card?	A) Yes	B) No			
11. Are you considering donating your organs after death?	A) Yes	B) No			

12. If your answer is no, what is the reason?	○ I don't feel ready	○ I have never thought about this	○ There is no specific reason	○ My health conditions are not suitable	○ I don't want my bodily integrity to be damaged
	○ I don't find it religiously appropriate	○ I don't know where to apply	○ I don't have enough information about organ donation	○ I don't want my organs to be taken while my heart is still working	○ I don't trust healthcare workers
	○ I think my organs will be used for commercial purposes	○ My family is against it	○ Other…………		

Part 2. ORGAN DONATION ATTITUDE SCALE

**Items**	**Strongly Disagree**	**Mostly Disagree**	**Partially Disagree**	**Slightly Agree**	**Somewhat Agree**	**Strongly Agree**
1. A person who wants to donate organs is considered a hero						
2. Organ donation disrupts the integrity of the body						
3. Donating a part of my body will ensure that a part of me continues to live even after my death						
4. Organ donors cannot have a say in which organs will be taken, even if specified beforehand						
5. We need a complete body for the afterlife (in the hereafter, in heaven)						
6. A person who agrees to donate their organs when they die sets a good example for others						
7. A person who decides to donate their organs when they die adds an extra goodness to their life						
8. My family would object to me signing an organ donation card						
9. Organ donation makes death more meaningful and valuable						
10. Declaring to donate your organs after death is a very moral behavior						
11. Doctors are not gentle with the human body when removing organs						
12. A person who agrees to donate their organs after death receives more respect and admiration from family and friends						
13. Preparing to be an organ donor brings to mind unpleasant thoughts about my own death						
14. When someone who has signed an organ donation card needs their life saved, all medical possibilities will not be used						
15. Donating organs is a way of giving thanks to God						
16. After a person signs an organ donation card, their chance of receiving adequate medical care will decrease						
17. It is highly likely that doctors will make an early death decision for someone who has signed an organ donation card						
18. Hearing about people whose lives are saved thanks to a new organ makes me think about the importance of donating my organs after death						
19. Organ donation should not be considered because the body is God's trust and has religious significance after death						
20. Donating organs after death is a way to ensure that some parts of the body are put to good use						
21. The surest way to cause my own death is to make plans for it, like signing an organ donor card						
22. Someone who allows part of their body to be transplanted to someone else offers a truly valuable gift						
23. Donating some parts of the body to those in need of organ transplantation when necessary is a humanitarian duty						
24. The death of an organ donor will be welcomed by doctors rather than treating them with great effort						
25. By agreeing to donate my organs after death, I give some people hope of survival						
26. It bothers me to give written permission for my organs to be taken and donated after death						
27. Organ donors are special people						
28. Organ donation is for the benefit of all humanity						
29. When I die, I want my entire body to die with me						
30. A person who intends to donate parts of their body when they die is highly likely to be declared dead despite being alive						
31. Life is too valuable to end because of an unhealthy heart or kidney, especially if the problem can be solved with organ donation						

32. When I sign an organ donation card, doctors can do something to me before I am completely dead						
33. A person carrying someone else's heart, eyes, or kidney is no longer like themselves						
34. By declaring that my organs can be taken after my death, I can enable someone else to live						
35. The thought of my body being cut and divided into parts after death makes me uncomfortable						
36. A person who declares that their organs can be taken after death can increase someone else's chance of recovery						
37. Despite special measures to protect the life of a person who has signed an organ donation card, there is still the possibility of attempting to take the life of the organ donor to save the life of a rich or very important person						
38. Donating an organ after my death would make me proud of myself						
39. When I die, I want to be buried whole with my own organs without my body being dismembered						
40. Declaring to donate organs is a sincere and selfless behavior						

Part 3. HEALTH LITERACY SCALE (HLS-14)

A. When reading instructions or brochures at hospitals or pharmacies, please select the appropriate option regarding whether you agree or disagree.	**Strongly disagree**	**Disagree**	**Undecided**	**Agree**	**Strongly agree**
1. I cannot read the materials I find.					
2. The text is written too small for me.					
3. The content is too difficult for me to understand.					
4. It takes me a long time to read them.					
5. I need someone's help to read them.					
B. If you are diagnosed with a disease and you don't have much knowledge about this disease and its treatment, please select the appropriate option regarding whether you agree or disagree.					
6. I gather information from various sources.					
7. I obtain the information I want.					
8. I understand the information I receive.					
9. I tell my doctor, family, or friends my own opinions about my illness.					
10. I apply the information I receive to my daily life.					
C. If you are diagnosed with a disease and acquire information about this disease and its treatment, please select the appropriate option regarding whether you agree or disagree.					
11. I consider whether the information is applicable to me.					
12. I consider whether the information is reliable.					
13. I check whether the information is valid and reliable.					
14. I gather information to implement my decisions about my own health.					

Part 4. FATALISM TENDENCY SCALE

Dear Participant;<br>Below are some thoughts on various topics. Please read them carefully. You are asked to determine your opinion on each thought and mark the option that corresponds to your opinion. There are no right or wrong answers to these thoughts; what matters is that you express your own thoughts sincerely.<br>Thank you for your interest and contributions.		**Strongly disagree**	**Disagree**	**Undecided**	**Agree**	**Strongly agree**
1.	Things that will happen in our lives are predetermined.					
2.	I think everything I experience is a result of my own behaviors.					
3.	I believe that some objects (evil eye bead, incense, lucky ring, etc.) are protective.					
4.	I believe that what will happen to me is my destiny.					
5.	I believe some objects bring bad luck.					
6.	I believe my future will be shaped according to the effort I show.					
7.	The unhappiness in people's lives is due to their bad luck.					
8.	The things we experience are a result of our choices.					
9.	I believe bad things come and find me.					
10.	I believe if I say certain words (jinn, etc.), I will be struck.					
11.	I believe I can direct some things with my own will.					
12.	If it's in my destiny, I will get sick.					
13.	I believe some people are lucky by birth.					
14.	The control of my life is in my hands.					
15.	No one can change their fate.					
16.	I think my destiny sometimes plays tricks on me.					
17.	I believe some rituals (knocking on wood, entering a room with the right foot, etc.) are protective.					
18.	When something I want doesn't happen, I say "it wasn't meant to be."					
19.	We go wherever our destiny throws us.					
20.	I believe I will be struck if I engage in certain behaviors.					
21.	People make their own choices.					
22.	I consent to my destiny directing my life.					
23.	I believe some situations (seeing a black cat, cutting nails at night, etc.) bring bad luck.					
24.	I think every job will happen as it's meant to be.					
